# Comment on ‘Contribution of pelvic and para-aortic lymphadenectomy with sentinel node biopsy in patients with IB2–IIB cervical cancer’

**DOI:** 10.1038/bjc.2012.225

**Published:** 2012-06-07

**Authors:** A Peres, A-l Margulies, E Barranger

**Affiliations:** 1 Department of Gynecology and Obstetrics, Lariboisiere Hospital, Diderot University Paris VII, AP-HP, 2 rue Ambroise-Pare, Paris 75010, France

**Sir,**

We read with great interest the article published by Chéreau *et al* (2012) demonstrating the interest of the detection of micrometastases in the locally advanced cervical cancers (LACC) by the sentinel node (SN) procedure. This study included 66 patients with LACC (FIGO stages IB2 to IIB). All patients underwent a pre-therapeutic pelvic and para-aortic lymphadenectomies before concurrent radiochemotherapy between 2002 and 2010. The SN procedure was performed in 45 patients.

The results of this retrospective study suggested that the disease-free survival (DFS) and the global survival (GS) were significantly altered when lymph node was metastatic in para-aortic area with or without metastatic pelvic lymph node.

The authors concluded that the SN procedure, by the increasing rate of occult metastases, would improve the selection of the patients who had a poor prognostic.

In spite of the interest of this study, some results deserve to be discussed:

First, in this study, the SN procedure has been proposed as a technique of ultrastaging of SN and not to avoid an unnecessary lymphadenectomy as suggested by some authors (Vicus and Covens, 2010).

Second, the low rates of identification (by patient and bilaterally), as well as the high rate of false negative, do not allow to retain this technique as reliable for LACC. Indeed, in this study, the rate of identification by patient was only 69% and the rate of the bilateral identification was only 26% with a high rate of false negative of 20%. These results were confirmed in other studies (Barranger *et al*, 2005), also showed a high rate of false negative of 20% and concluded that the SN biopsy is reliable in early-stage cervical cancer but not in locally advanced disease.

Third, the authors showed that 20% of patients with a lymph node involvement were diagnosed, only thanks to the ultrastaging of the SN procedure. All the SNs in this study were distributed only in the pelvic area. According to them, the pelvic lymph node involvement had no significant impact on DFS and GS. So, the authors could not conclude that the increase of the detection of the micrometastases with ultrastaging could improve the knowledge of the prognostic because the prognostic depends on the para-aortic node status according to their results.

Besides, the prognostic value of the micrometastases for LACC and adjuvants therapy for these patients is still controverted in the literature and remains a subject of debate. Horn *et al* (2008) confirmed that the micrometastatic disease represented an independent prognostic factor but they only studied the pelvic area. The majority of the data have been evaluated in early-stage disease and only in pelvis lymph nodes (Fregnani *et al*, 2006; Horn *et al*, 2008).

In conclusion, we think that there is no place for the SN procedure in the management of LACC. Indeed, according to these results, ultrastaging has no impact on DFS and GS.

## References

[bib1] Barranger E, Coutant C, Cortez A, Uzan S, Darai E (2005) Sentinel node biopsy is reliable in early-stage cervical cancer but not in locally advanced disease. Ann Oncol 16: 1237–1242 15890666 10.1093/annonc/mdi245

[bib2] Chéreau E, Feron JG, Ballester M, Coutant C, Bezu C, Rouzier R, Touboul E, Darai E (2012) Contribution of pelvic and para-aortic lymphadenectomy with sentinel node biopsy in patients with IB2-IIB cervical cancer. Br J Cancer 106: 39–44 22146520 10.1038/bjc.2011.541 PMC3251874

[bib3] Fregnani JH, Latorre MR, Novik PR, Lopes A, Soares FA (2006) Assessment of pelvic lymph node micrometastatic disease in stages IB and IIA of carcinoma of the uterine cervix. Int J Gynecol Cancer 16: 1188–1194 16803505 10.1111/j.1525-1438.2006.00519.x

[bib4] Horn LC, Hentschel B, Fischer U, Peter D, Bilek K (2008) Detection of micrometastases in pelvic lymph nodes in patients with carcinoma of the cervix uteri using step sectioning: frequency, topographic distribution and prognostic impact. Gynecol Oncol 111: 276–281 18722005 10.1016/j.ygyno.2008.07.017

[bib5] Vicus D, Covens A (2010) Role of sentinel lymph node biopsy in cervical cancer: pro. Int J Gynecol Cancer 20: S34–S36 20975360 10.1111/IGC.0b013e3181f60d60

